# Efficacy and tolerability of brexpiprazole in patients with psychotic and mood disorders: a pilot study

**DOI:** 10.1192/j.eurpsy.2022.881

**Published:** 2022-09-01

**Authors:** S.C. Civardi, N. Bassetti, V. Arienti, F. Besana, P. Politi, N. Brondino, M. Olivola

**Affiliations:** University of Pavia, Department Of Brain And Behavioral Sciences, Pavia, Italy

**Keywords:** Brexpiprazole, psychopharmacology, Mood disorders, PSYCHOTIC DISORDERS

## Abstract

**Introduction:**

Brexpiprazole is a novel antipsychotic drug. It exerts antagonistic activity at the serotonin 5HT2A, 5HT2B, 5HT7 and noradrenaline alpha 1b/2c receptors; it also acts as partial agonist of serotonin 5HT1 and dopamine D2, D3 receptors. Brexpiprazole is approved for the treatment of schizophrenia and as an add-on therapy for major depression.

**Objectives:**

This pilot study aims at exploring efficacy and tolerability of Brexpiprazole in a small sample of patients diagnosed with either a psychotic or a mood disorder.

**Methods:**

This observational study was conducted at our Acute Psychiatric Inpatient Unit. We included 7 patients (5 males, 2 females) hospitalized between 2020 and 2021, diagnosed with schizophrenia spectrum disorders or mood disorders with psychotic symptoms confirmed by Mini International Neuropsychiatric Interview. Patients who participated signed an informed consent. Information concerning diagnosis, demographic characteristics (age, sex, education, marital status) and pharmacological therapy were collected examining clinical records. The average lenght of hospitalization was 13.4 days. Psychopathology was assessed by means of the PANSS and the severity of the illness was evaluated with CGI severity scale (CGI-S), both on admission and discharge. We also administered the UKU scale to evaluate the tolerability profile.

**Results:**

**Figure:**
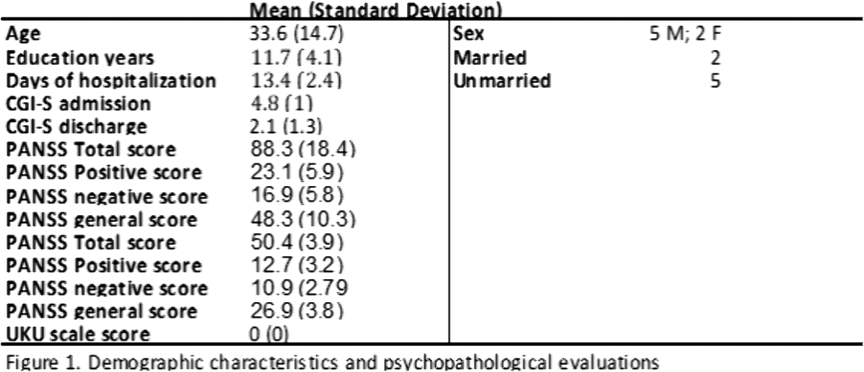

**Figure:**
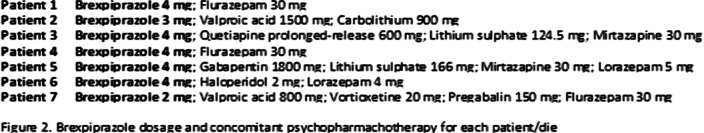

.

**Figure:**
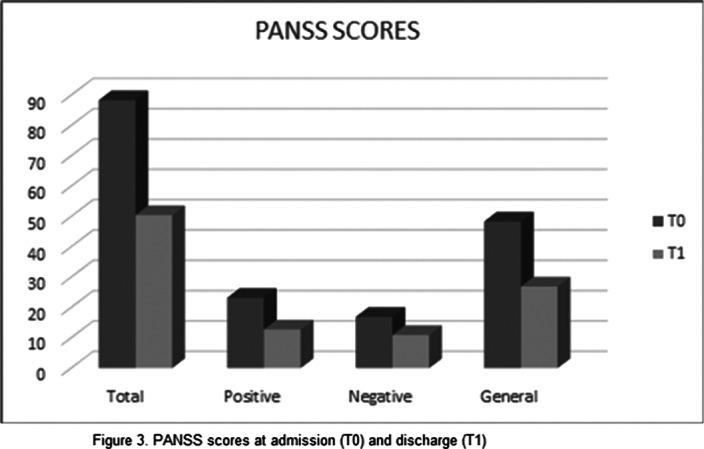

Results can be seen in figures 1, 2, 3

**Conclusions:**

Our study found a significant improvement in both positive and negative symptoms, with good tolerability. Limitations of our study are: small sample size and limited period of observation. These premises suggest that further research is needed in order to elucidate the exact mechanisms underlying Brexpiprazole’s action and the possible implication in mood disorders.

**Disclosure:**

No significant relationships.

